# Case Report: Clinical manifestations and treatment of two Chinese patients with FINCA syndrome carrying a novel variant of *NHLRC2*

**DOI:** 10.3389/fped.2024.1402545

**Published:** 2024-09-12

**Authors:** Yuemei Liu, Hongling Wang, Yu Tang, Lei Zhang, Yanyan Su, Yanqion Wang, Shasha Xu, Shiyue Mei, Chunyang Jia, Yuelin Shen, Xiaolei Tang

**Affiliations:** ^1^ Department of Respiratory Medicine, Children’s Hospital Affiliated to Zhengzhou University, Henan Children’s Hospital, Zhengzhou Children’s Hospital, Zhengzhou, China; ^2^ Neonatology Department, Maternity and Child Health Care Hospital of Rizhao, Rizhao, China; ^3^ Henan Key Laboratory of Pediatric Genetics and Metabolic Diseases, Children’s Hospital Affiliated to Zhengzhou University, Henan Children’s Hospital, Zhengzhou Children’s Hospital, Zhengzhou, China; ^4^ Radiology Department, Children’s Hospital Affiliated to Zhengzhou University, Henan Children’s Hospital, Zhengzhou Children’s Hospital, Zhengzhou, China; ^5^ Department of Respiratory Medicine, Beijing Children’s Hospital, National Center for Children’s Health, Capital Medical University, Beijing, China

**Keywords:** *NHLRC2*, interstitial lung disease, lung fibrosis, neurodegeneration, genetics

## Abstract

Fibrosis, neurodegeneration, and cerebral angiomatosis (FINCA) syndrome is an autosomal recessive genetic disorder caused by mutations in NHL-repeat-containing protein 2 (*NHLRC2*) gene. This case report describes two Chinese siblings with FINCA syndrome carrying a novel frameshift variant, c.1610dupT (p.L537Ffs*17), of *NHLRC2* gene. They shared similar symptoms of interstitial lung disease (ILD) and neurodegeneration, with early onset during infancy, and shared similar chest CT findings of bilateral ground-glass opacities and consolidations. The elder brother died of infantile respiratory failure, while the younger brother showed improvement in respiratory symptoms, chest CT, and Krebs von den Lungen-6 levels after long-term systemic glucocorticoid therapy, indicating that anti-inflammatory treatment may be beneficial in the treatment of ILD caused by FINCA syndrome.

## Introduction

Fibrosis, neurodegeneration, and cerebral angiomatosis (FINCA) syndrome, an autosomal recessive disorder caused by a variant of the NHL-repeat-containing protein 2 (*NHLRC2*) gene, characterized by interstitial lung fibrosis, neurodegeneration, and cerebral angiomatosis (1). FINCA syndrome may cause early infant death, mainly due to respiratory failure caused by progressive interstitial lung disease (ILD). However, no effective treatment has been reported currently. In this article, we report two Chinese siblings carrying a novel *NHLRC2* gene variant. The ILD responded well to long-term systemic glucocorticoid therapy in the younger brother.

## Case report

**Figure 1:**
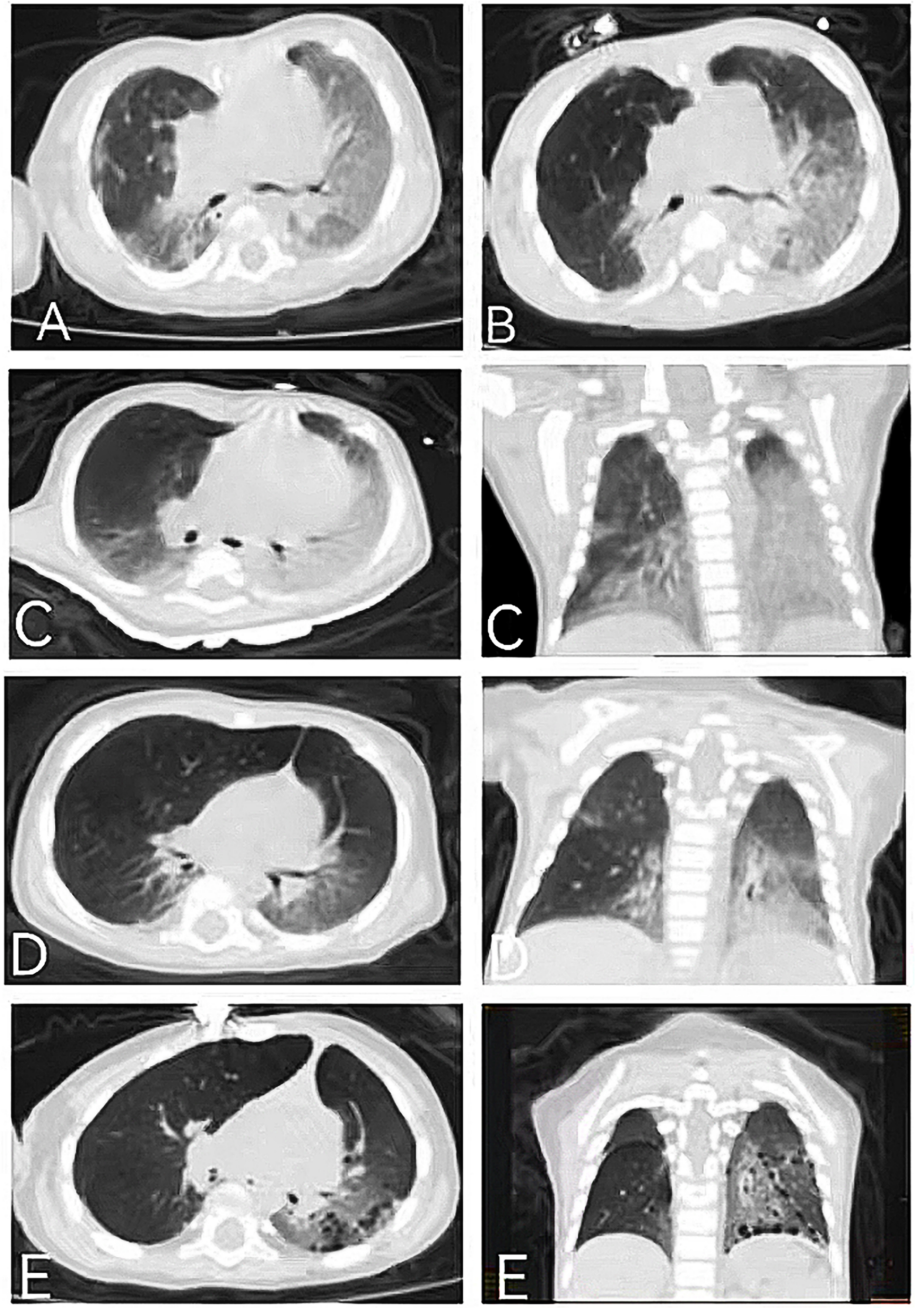
Chest CT of the elder **(A**,**B)** and younger **(C**–**E)** brothers: **(A)** chest CT of the elder brother showing bilateral GGOs, consolidations, and reticular opacities at the age of 3 months; **(B)** chest CT of the elder brother showing slightly improved GGOs but aggravated consolidations at the age of 8 months; **(C)** chest CT of the younger brother showing GGOs, consolidations, and mediastinal lung hernia at the age of 1 month; **(D)** chest CT of the younger brother showing improved GGOs and consolidations at the age of 4 months; and **(E)** chest CT of the younger brother showing the appearance of cysts and improved GGOs and consolidations at the age of 14 months.

These two patients were siblings. Case 1 was the elder brother, a full-term boy with a birth weight of 2.8 kg and congenital heart defects (coarctation of the aorta, ventricular septal defect, atrial septal defect, and patent ductus arteriosus). He underwent a cardiac operation at the age of 1 month and also developed neonatal jaundice. He presented with recurrent cough, wheezing, and tachypnea beginning at 2 months. He has also experienced developmental delay, recurrent diarrhea, feeding problems, and poor weight gain from the age of 2 months. During illness, he needed nasogastric feeding but was fed orally after discharge. He had two episodes of respiratory exacerbations accompanied by respiratory failure, leading to hospitalizations at the ages of 3 and 8 months. During his first hospitalization at 3 months, a physical examination revealed crackles, wheezing, retractions, funnel chest, hypotonia, and poor visual contact. His respiratory rate was 60–90 times/min, and his oxygen saturation (SpO_2_) was 80%–92%, requiring oxygen supplementation. He was anemic (hemoglobin 73 g/L), and his liver function indicators were slightly elevated [alanine aminotransferase (ALT) 53 U/L, total bilirubin (TBIL) 22 μmol/L]. His white blood cell count, C-reactive protein (CRP), erythrocyte sedimentation rate (ESR), renal function, and thyroid function tests were within normal ranges. *Pneumocystis jirovecii* were found in the bronchoalveolar lavage fluid. Immunological work-up showed a mild decrease in IgG and IgA levels, while lymphocyte subtypes remained normal. A chest CT showed bilateral ground-glass opacities (GGOs) and consolidations at the age of 3 months (Figure 1A). He developed respiratory failure necessitating invasive ventilation for 6 days and received anti-infection treatment for 1 month, with multiple antibiotics including trimethoprim sulfamethoxazole for *P. jirovecii* and antifungal drugs. He also received prednisone at a daily dose of 0.75 mg/kg for 20 days. After 1 month, repeated chest CT showed little improvement. After discharge, his cough improved, but tachypnea and oxygen desaturation persisted (respiratory rate 50–60 times/min, SpO_2_ 94%). At the age of 8 months, he presented with a short-term fever followed by another episode of respiratory failure. Multiple pathogens were found in the aspiration of sputum obtained from the oropharynx, nasal cavity, and bronchoalveolar lavage fluid during the second hospitalization, including *Staphylococcus aureus*, *Moraxella catarrhalis*, *Candida parapsilosis*, rhinovirus, and human metapneumovirus. The repeated chest CT indicated GGOs in the same area as before and increased consolidations (Figure 1B). Unfortunately, despite anti-infection treatment, he died of respiratory failure at the age of 8 months.

**Figure 2:**
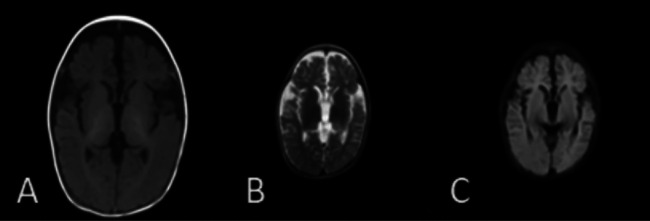
Brain MRI of the younger brother [**(A)** T1, **(B)** T2, **(C)** T2 FLAIR]: brain MRI of the younger brother revealing a widening of cerebral sulci and fissures, cortical thinning, and enlargement of the frontal and temporal angles at the age of 14 months.

Case 2 was the younger brother, a full-term boy with a birth weight of 2.85 kg, who also developed neonatal jaundice. From the age of 1 month, he developed symptoms similar to his brother, including recurrent cough, tachypnea, developmental delay, recurrent diarrhea, and poor weight gain. He was able to feed orally without swallowing disorders. A physical examination revealed crackles, retractions, hypotonia, and poor visual contact. His respiratory rate was 50–80 times/min, and his SpO_2_ was 90%–92%, requiring oxygen supplementation during hospitalization. He was anemic (hemoglobin 69 g/L), and his liver function indicators were slightly elevated (ALT 103 U/L, TBIL 51.5 μmol/L). No pathogens, including *P. jirovecii*, viruses, bacteria, and fungi, were found during hospitalization. Autoantibody testing indicated positive perinuclear anti-neutrophil cytoplasmic antibodies (p-ANCAs). Krebs von den Lungen-6 (KL-6), a biomarker for ILD activity, was elevated at 2,107 U/ml (normal range <500 U/ml). His chest CT was similar to his brother's, showing GGOs and consolidations, mainly distributed in the lower lungs (Figure 1C). At the age of 1 month, steroid treatment was started (daily dose of 2–1 mg/kg of methylprednisolone for 2 weeks, followed by oral prednisone with a tapered dosage from 0.7 to 0.2 mg/kg/day in 2 months and then maintained at 0.2 mg/kg/day for 1 year), combined with short-term antibiotics for prevention of infection. After more than 2 months of treatment (at the age of 4 months), his chest CT improved (Figure 1D), with repeated KL-6 levels decreasing to 1,417 U/ml. After 13 months of treatment (at the age of 14 months), his chest CT showed the development of cysts and improvement in GGOs and consolidations (Figure 1E). Repeated KL-6 levels decreased to 430 U/ml. His respiratory symptoms, such as cough and tachypnea, improved. His respiratory rate was 30–40 times/min, and SpO_2_ was maintained at 97%–100% at the age of 14 months. He did not require oxygen supplementation at home. However, his neurological symptoms did not alleviate. At the age of 4 months, his development quotient (DQ) evaluated by the Chinese Development Scale for Children Aged 0–6 years was 39 (normal range >85), indicating moderate intellectual disability. He experienced his first seizure at 7 months with no inducement, followed by another seizure at 14 months of age, accompanied by vomiting and diarrhea. He did not receive antiepileptic therapy. At the age of 14 months, his motor development was significantly delayed compared to his peers. He could not turn over, crawl, or walk. His brain magnetic resonance imaging (MRI) showed a widening of cerebral sulci and fissures, cortical thinning, and enlargement of the frontal and temporal angles, without evidence of cerebral angiomatosis (Figures 2A–C). He continued to be fed orally at home and weighed 7 kg.

**Figure 3:**
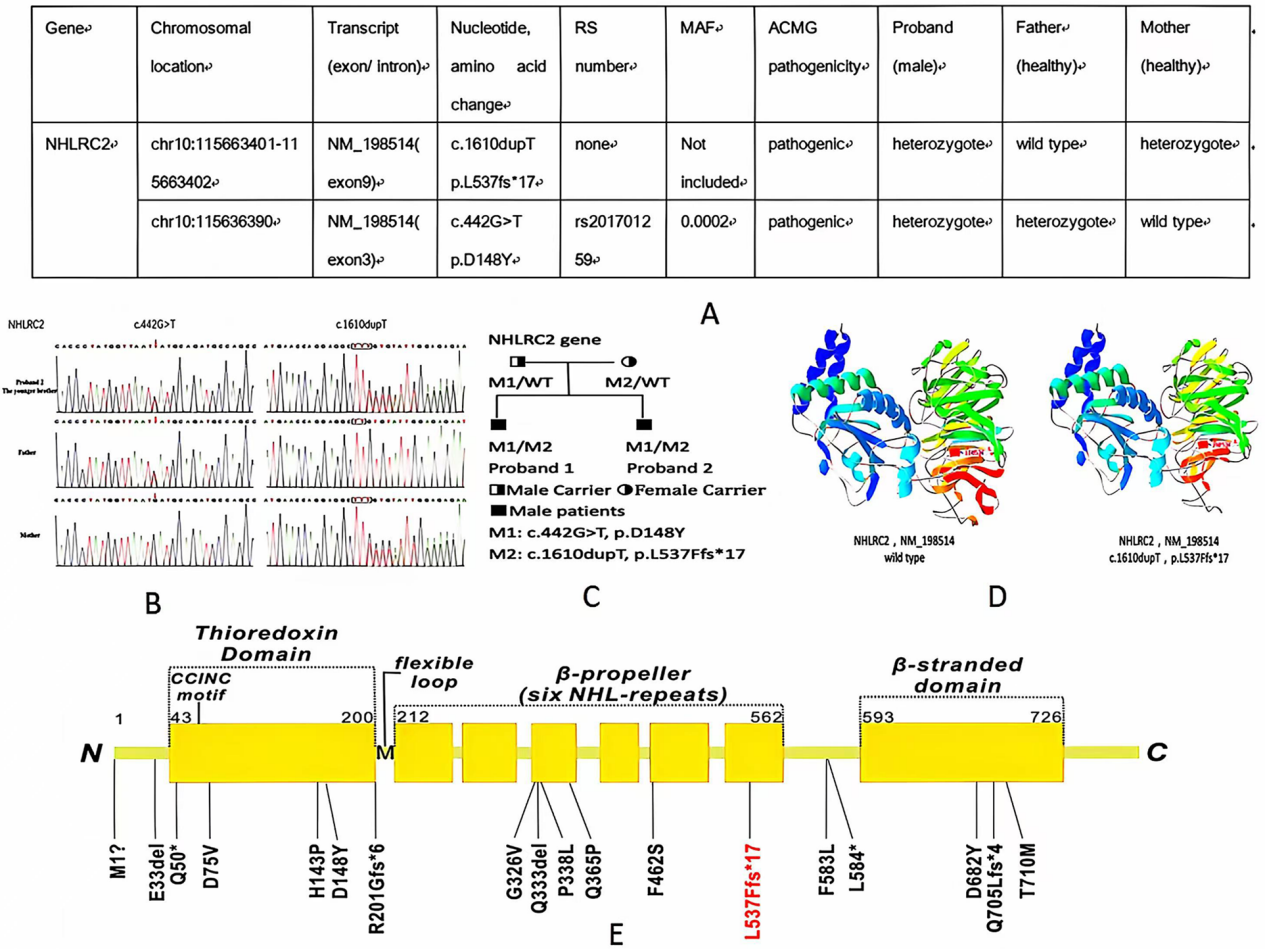
**(A**,**B)** WES information and Sanger sequencing of *NHLRC2* variants of the elder and younger brothers; **(C)** pedigree of the family; **(D)** structure of the *NHLRC2* wild-type and c.1610dupT, p.L537Ffs*17 variant; and **(E)** novel variant (red color) and previously reported variants of *NHLRC2* (black color) 1, 8–11.

Whole-exome sequencing (WES) was performed in both of the siblings, showing the same findings of two compound heterozygous variants of *NHLRC2* gene. One variant, c.442 G>T, p.D148Y, was inherited from the father. The other, a novel frameshift variant, c.1610dupT, p.L537Ffs*17, was inherited from the mother. The results were validated by Sanger sequencing (Figures 3A–C). The p.D148Y variant is a missense mutation, which has been previously reported in the Human Gene Mutation Database (HGMD) with associated evidence of pathogenicity, and the ClinVar database classifies this variant as pathogenic. PCR Sanger sequencing validated that the probands’ father is heterozygous for this mutation, while the probands’ mother does not carry the mutation. According to the standards and guidelines set by the American College of Medical Genetics and Genomics (ACMG), this variant is pathogenic. The other novel variant, p.L537Ffs*17, is a frameshift mutation leading to a truncated protein (Figure 3D), which qualifies as pathogenic very strong (PVS1) evidence of ACMG criteria. PCR Sanger sequencing verified that the probands’ father does not have this mutation, while the probands’ mother is heterozygous for it, forming a compound heterozygous mutation in combination with the p.D148Y variant. The inheritance pattern is consistent with autosomal recessive inheritance, and both affected brothers in the family carry the same variants, exhibiting similar symptoms of ILD and neurological involvement, which aligns with the clinical phenotype of FINCA syndrome caused by *NHLRC2* mutations, constituting PM2 supporting evidence. This variant is exceedingly rare in the general population, with no record in normal population databases, and there are no reports in either the HGMD or ClinVar databases, which constitutes PM3 evidence. Therefore, based on ACMG criteria, the p.L537Ffs*17 variant of *NHLRC2* gene is predicted to be pathogenic. Protein structure analysis of *NHLRC2* showed that the p.L537Ffs*17 variant leads to an incomplete protein structure beyond the sixth NHL-repeat in the β-propeller domain (Figure 3D).

## Discussion

FINCA syndrome is a recently discovered monogenetic disease related to NHLRC2 dysfunction. Lung fibrosis, neurodegeneration, and cerebral angiomatosis are primary manifestations of FINCA syndrome. NHLRC2 is a 79-kDa protein composed of 726 amino acids containing three domains, an N-terminal thioredoxin-like (Trx-like) domain, a six-bladed NHL-repeat-containing β-propeller domain, and a C-terminal β-stranded domain (2). Downregulation of NHLRC2 has been shown to increase the susceptibility of human colon cancer cells to reactive oxygen species (ROS)–induced apoptosis (3). NHLRC2 also plays an important role in phagocytosis by controlling actin polymerization, filopodium formation (4), and T-cell homeostasis (5). The pathogenic mechanisms leading to the clinical manifestations of ILD/lung fibrosis and neurodegeneration in FINCA syndrome caused by NHLRC2 deficiency have not yet been fully elucidated. NHLRC2 has been previously flagged in a study as a differentially expressed gene when comparing rapidly and slowly progressing idiopathic pulmonary fibrosis (IPF) patients (6). It also has been linked to decreased lung function values (7). A study conducted by Paakkola et al. suggested that NHLRC2 may induce tissue fibrosis (8). NHLRC2 was identified to interact in several cytosolic processes, including cell–cell adhesion, cell division, and intracellular protein transport using proximity-labeling mass spectrometry. A transmission electron microscopy analysis of immortalized cell cultures derived from skin biopsies of FINCA patients demonstrated multilamellar bodies and distinctly organized vimentin filaments. In addition, in two out of three cultures from patient-derived skin biopsies, cells displaying characteristics typical of myofibroblasts were identified. These findings suggested *NHLRC2* induces severe tissue fibrosis by enhancing the differentiation of fibroblasts into myofibroblasts, regulating the cytoskeleton, and affecting vimentin intermediate-size filaments, vesicle transportation, and pro-inflammatory regulators (8). In another study, Hiltunen et al. made a proteomic analysis of the *NHLRC2* FINCA mice model harboring the missense mutation p.(D148Y) of a FINCA patient. Compared to wild-type mice, FINCA mice revealed dysfunction in vesicular trafficking. According to the authors, *NHLRC2* dysfunction is associated with the accumulation of RNA-binding proteins in a FINCA mouse model, suggesting that disrupted RNA metabolism may contribute to neurodegeneration in FINCA patients (9). In future research, metabolomics and proteomics studies may play an active role in identifying biomarker signatures related to disease phenotypes and monitoring therapeutic interventions.

So far, only 17 variants from 29 patients of NHLRC2 have been identified to be associated with FINCA syndrome (1, 10–14) (Figure 3E). The p.D148Y variant is a hotspot variant found in most patients with FINCA syndrome, including our newly reported patients. The p.D148Y variant is located in the Trx-like domain, which is a characteristic of oxidoreductases and thiol–disulfide exchange (2). To investigate for a possible genotype–phenotype correlation, Sczakiel et al. observed a correlation of remaining *NHLRC2* protein levels with phenotype severity. They speculate that variants leading to severely reduced protein levels (either in a homozygous state or in a compound heterozygous state with another severe missense or frameshift/nonsense variant) are associated with an early-onset multisystem phenotype that includes pulmonary disease (14). In our study, we reported a novel variant of c.1610dupT, p.L537Ffs*17 in two patients from one family, which is the frameshift variant. It is located in the sixth NHL-repeat in the β-propeller domain, which functions as a protein–protein interaction module (15) (Figure 3E). Both siblings presented with early onset and severe respiratory and neurological symptoms, with one succumbing to respiratory failure in infancy, indicating that the p.L537Ffs*17 variant may be associated with severe phenotypes.

Among the 31 FINCA patients, including our newly reported patients, 14 patients (45.2%) were boys (1, 10–14). All FINCA patients experienced neurological symptoms including developmental delay, intellectual disability, behavior problems, movement disorder, hypotonia, dystonia, and seizures, among others (1, 10–14). Other clinical manifestations involve the respiratory system, such as ILD; gastrointestinal issues like diarrhea, hepatomegaly, feeding problems, and liver dysfunction; cardiovascular complications including congenital heart disease, cardiomegaly, pulmonary hypertension, and dilation of the ascending aorta; and other system involvements, such as anemia and renal insufficiency (1, 10–14) (as presented in Table 1). About half of the 31 FINCA patients presented with respiratory symptoms, such as tachypnea, cough, hypoxemia, and respiratory distress. Chest CT scans were performed on 11 patients, among whom 9 children were diagnosed with ILD, with the most prevalent findings being GGOs and pulmonary consolidations. It is noteworthy that fatalities predominantly occur in patients with the ILD phenotype. The overall mortality rate among 31 FINCA patients is 25.8%, whereas the mortality rate for those 9 patients with comorbid ILD is significantly higher at 77.8%. All deceased children died before the age of 3 (1, 10–14). This suggests that ILD is a significant factor contributing to the early mortality of pediatric FINCA patients, necessitating particular attention.

**Table 1.** Clinical manifestation and genotype of thirty-one patients with FINCA syndrome.

Individual (Family)	proband1 (F1)	proband2 (F1)	proband3^1^ (F2)	proband4^1^ (F2)	proband5^1^ (F3)	proband6^8^ (F4)	proband7^9^ (F5)	proband8^9^ (F5)	proband9^9^ (F5)	proband10^9^ (F6)	proband11^9^ (F7)	proband12^9^ (F7)	proband13^10^ (F8)	proband14^10^ (F9)	proband15^10^ (F10)	proband16^11^ (F11)
Sex	male	male	male	male	male	male	male	female	female	female	male	female	female	female	female	male
Genetic variants	p.D148Y/ p.L537Ffs*17	p.D148Y/ p.L537Ffs*17	p.D148Y/p.R201Gfs*6	p.D148Y/p.R201Gfs*6	p.D148Y/p.R201Gfs*6	p.D148Y/p.H143P	N/A	p.D148Y/p.D75V	p.D148Y/p.D75V	p.D148Y/p.P338L	Homo p.D148Y	Homo p.D148Y	Homo p.D148Y	Homo p.D148Y	p.D148Y/p.G326V	p.F583L/p.T710M
Origin	Chinese	Chinese	Finland	Finland	Finland	Ukrainian	Greek	Greek	Greek	Belgian	Jordanian	Jordanian	Polish	Polish	Polish	Chinese
Duration of gestation	full term	full term	full term	full term	36+1weeks	full term	full term	full term	full term	full term	full term	full term	N/A	N/A	N/A	full term
Birth weight	2800 g	2850 g	3690 g	3280 g	2910 g	3050 g	N/A	N/A	N/A	N/A	N/A	N/A	N/A	N/A	N/A	3700 g
Apgar score	10,10	10,10	10,10	9,9	10,10	8,8	N/A	N/A	N/A	N/A	N/A	N/A	N/A	N/A	N/A	N/A
Age of onset	2 m	1 m	2 m	2 m	0-2 m	0 m	7 m	5 m	0-2 m	0.5m	0 m	12 m	0 m	9 m	3 m	17d
Respiratory involvements	+	+	+	+	+	+	+	+	+	+	+	−	N/A	N/A	N/A	+
ILD	+	+	+	+	+	+	+	+	−	+	N/A	N/A	N/A	N/A	N/A	N/A
^a^Neurological involvements	+	+	+	+	+	+	+	+	+	+	+	+	+	+	+	+
^b^Gastrointestinal involvements	+	+	+	+	+	+	+	+	+	+	N/A	N/A	−	−	+	N/A
^c^Cardiovascular involvements	+	−	+	−	−	+	−	−	−	+	−	−	−	−	−	+
Anemia	+	+	+	+	+	+	+	−	−	+	−	−	−	+	−	N/A
Kidney dysfunction	−	−	+	−	−	−	N/A	N/A	N/A	N/A	N/A	N/A	N/A	N/A	N/A	N/A
Immune system	decreased IgG and IgA	N/A	N/A	N/A	decreased IgG	decreased IgG, decreased CD3, CD4, CD8 cells	decreased IgG and IgA	decreased IgG	−	decreased IgG	N/A	N/A	N/A	N/A	N/A	N/A
Autoantibodies	N/A	p-ANCA(+)	N/A	N/A	N/A	N/A	N/A	N/A	N/A	N/A	N/A	N/A	N/A	N/A	N/A	N/A
Age at last follow-up	8 m	14 m	21 m	13 m	14 m	29 m	22 m	17 m	10 y	4 y	14 y	7 y	6 y	9 y	12 y	27 d
Status at last follow-up	death	alive	death	death	death	death	death	death	alive	alive	alive	alive	alive	alive	alive	death

Individual (Family)	proband17^14^ (F12)	Proband18^14^ (F12)	proband19^14^ (F13)	Proband20^14^ (F14)	Proband21^14^ (F15)	Proband22^14^ (F16)	Proband23^14^ (F17)	Proband24^14^ (F17)	Proband25^14^ (F18)	Proband26^14^ (F19)	Proband27^14^ (F20)	Proband28^14^ (F21)	Proband29^14^ (F21)	Proband30^14^ (F22)^17^	Proband31^14^ (F23)
Sex	female	male	female	female	female	Female	male	male	male	male	female	female	female	female	female
Genetic variants	Homo p.M1?	Homo p.M1?	Homo p.D148Y	p.L584*/ p.D692Y	p.Q50*/ p.D148Y	p.Q365P/ p.F462S	Homo p.M1?	Homo p.M1?	Homo p.D148Y	Homo p.E33del	p.Q333del/ p.Q705Lfs*4	Homo p.D148Y	Homo p.D148Y	Homo p.D148Y	Homo p.D148Y
Origin	Syria		Palestine	Belgian, Hungarian, English, and Scottish	German	African American, Scandanavian, Irish, English, German	Libanon		Iran	Iran	Caucasian	Iran		Pakistan	Iran
Duration of gestation	full term	N/A	full term	full term	full term	full term	full term	full term	36weeks	full term	full term	full term	N/A	full term	full term
Birth weight	3200 g	3300 g	2800 g	3033g	3300 g	3005 g	N/A	N/A	2900 g	3300 g	N/A	3200 g	3000 g	N/A	3520
Apgar score	N/A	N/A	N/A	N/A	N/A	N/A	N/A	N/A	N/A	N/A	N/A	N/A	N/A	N/A	N/A
Age of onset	N/A	N/A	N/A	N/A	N/A	N/A	N/A	N/A	N/A	N/A	N/A	N/A	N/A	N/A	N/A
Respiratory involvements	−	−	−	−	+	−	−	−	−	+	−	−	−	−	−
ILD	−	−	−	−	+	−	−	−	−	+	−	−	−	−	−
^a^Neurological involvements	+	+	+	+	+	+	+	+	+	+	+	+	+	+	+
^b^Gastrointestinal involvements	−	−	−	+	+	−	−	−	−	+	−	−	−	−	+
^c^Cardiovascular involvements	−	−	−	−	+	−	−	−	−	−	+	−	−	−	−
Anemia	+	−	−	−	+	−	−	−	−	+	−	+	−	−	−
Kidney dysfunction	−	−	−	−	−	−	−	−	−	−	+	−	−	−	−
Immune system	N/A	N/A	N/A	N/A	N/A	N/A	N/A	N/A	N/A	N/A	N/A	N/A	N/A	N/A	N/A
Autoantibodies	N/A	N/A	N/A	N/A	N/A	N/A	N/A	N/A	N/A	N/A	N/A	N/A	N/A	N/A	N/A
Age at last follow-up	5 y	19 y	2 y	15 y	22 m	13 y	19 y	9 y	8 y	5 y	8 y	·4 y	10 y	7 y	6 y
Status at last follow-up	alive	alive	alive	alive	dead	alive	alive	alive	alive	alive	alive	alive	alive	alive	alive

m: months; y: years; d: days; Homo: homozygous; N/A, not available; ILD, interstitial lung disease; ANCA, antineutrophil cytoplasmic antibodies. ^a^Neurological involvements: include developmental delay, intellectual disability, behavior problem, movement disorder, hypotonia, dystonia and seizures; ^b^Gastrointestinal involvements: include diarrhea, hepatomegaly, feeding problem and liver dysfunction; ^c^Cardiovascular involvements: include congenital heart disease, cardiomegaly, pulmonary hypertension, and dialation of ascend aorta.

Histological findings in the lung of ILD patients, based on lung biopsy or autopsy, include granuloma-like lesions surrounded by myofibroblasts, non-specific interstitial pneumonitis (NSIP), cholesterol pneumonitis, desquamative interstitial pneumonia (DIP), diffuse alveolar damage (DAD), and pulmonary alveolar proteinosis (PAP) (1, 10–12) (Table 2). These symptoms, along with chest CT and lung histological findings, are not specific to ILD and may remind us of surfactant dysfunction disorders. For example, the clinical manifestations of surfactant dysfunction disorder caused by an *NKX2-1* mutation are very similar to those of early-onset ILD and neurological involvement, which needs to be excluded as a differential diagnosis. Unlike in the *NKX2-1* mutation, FINCA patients exhibit normal thyroid function. Other diseases, such as STING-associated vasculopathy with onset in infancy (SAVI) and COPA syndrome, which may present with early-onset ILD and occasionally accompanied by neurological involvement, and FLNA mutations, which may cause ILD, skeletal dysplasia, neuronal migration abnormality, cardiovascular malformation, intellectual disability, and intestinal obstruction (16), should also be excluded as differential diagnoses.

**Table 2:** Respiratory symptoms, treatments, and prognosis of nine patients with FINCA syndrome associated with ILD.

Individual (family)	Proband1 (F1)	Proband2 (F1)	Proband3^1^ (F2)	Proband4^1^ (F2)	Proband5^1^ (F3)	Proband6^8^ (F4)	Proband7^9^ (F5)	Proband8^9^ (F5)	Proband10^9^ (F6)
Tachypnea	+	+	+	+	+	+	+	+	+
Cough	+	+	N/A	N/A	+	N/A	N/A	N/A	N/A
Hypoxemia	+	+	−	+	+	N/A	+	+	+
Recurrent respiratory infections	+	+	+	+	+	+	+	+	+
Mechanical ventilation	+	−	N/A	N/A	N/A	+	+	+	−
Pathogens	*P. jirovecii*, *S. aureus*, *M. catarrhalis*, rhinovirus, human metapneumovirus, *C. parapsilosis*	−	−	N/A	Influenza B	Bacterial and viral	−	N/A	−
Chest HRCT	GGOs, consolidations	GGOs, consolidations, cysts, and mediastinal lung hernia	Consolidations and reticular opacities	GGOs, interstitial infiltration, atelectasis, enlarged thymus and left hilar	GGOs, interstitial septal thickening, and lobular pleural thickening	Consolidations/atelectasis, air bronchograms, compressed trachea, and pectus excavatum	GGOs and consolidations	GGOs, consolidations, mosaic patterns, bronchiectasis, interstitial and alveolar markings, and cysts	GGOs, paraseptal and centrilobular emphysema, and cysts
Lung histology	N/A	N/A	NSIP, DAD, granuloma-like lesions surrounded by myofibroblasts	NSIP, granuloma-like lesions surrounded by myofibroblasts	NSIP, DAD, granuloma-like lesions surrounded by myofibroblasts	Fibrosis	DIP with diffuse alveolar damage	PAP	Cholesterol Pneumonitis, NSIP
Treatment for ILD	Short-term steroids and antibiotics	Long-term steroids	N/A	N/A	N/A	N/A	Steroids, catecholamines, and inhaled nitric oxide	Hydroxychloroquine and steroids	Hydroxychloroquine, pulse corticosteroids
Response to steroids	±	+	N/A	N/A	N/A	N/A	−	−	±
Age at the last follow-up	8 months	14 months	21 months	13 months	14 months	29 months	22 months	17 months	4 years
Status at the last follow-up	Death	Alive	Death	Death	Death	Death	Death	Death	Alive

GGOs, ground-glass opacities; NSIP, non-specific interstitial pneumonia; DAD, diffuse alveolar damage; DIP, desquamative interstitial pneumonia; PAP, pulmonary alveolar proteinosis; N/A, not available; ILD, interstitial lung disease; HRCT, high-resolution computed tomography.

Until now, there have been few reports on the treatment of ILD caused by FINCA syndrome. Progressive respiratory symptoms and exacerbations due to ILD were the main causes of death in the previously reported cases. Therefore, the treatment of ILD contributes significantly to the prognosis of FINCA syndrome. Systemic glucocorticoids, the first-line treatment for ILD, have been administered to four patients with FINCA syndrome before. However, the efficacy of the treatment remains uncertain (11). Two of them survived. One of them responded well to high-dose steroids. The other patient received anti-inflammatory treatment, including glucocorticoids and hydroxychloroquine, until the age of 2 years, and the patient's medical condition stabilized over the first 3 years of life (11). Two patients died despite glucocorticoid treatment, while the duration of their treatment remains unclear (11) (as presented in Table 2). In our case study, we reported a patient with FINCA syndrome undergoing long-term steroid treatment since the age of 1 month. After 13 months of treatment, the patient’s respiratory symptoms improved, KL-6 levels decreased, and chest CT showed improvement, indicating that long-term glucocorticoids or other anti-inflammatory therapies may be beneficial in the treatment of FINCA-induced ILD. Although the physiological function of *NHLRC2* is not yet fully elaborated, previous studies have demonstrated that the possible pathogenic mechanism of *NHLRC2* is associated with inflammation or autoimmunity. Overexpression of *NHLRC2* has been shown to decrease the expression levels of vimentin and IL-1β, suggesting that *NHLRC2* deficiency may be involved in the mechanism of fibrogenesis by regulating inflammatory pathways (8). In addition, *NHLRC2* expression was found to increase under inflammatory conditions in an equine model of chronic asthma (17). Zinc finger and AT-hook domain containing (Zfat) protein, a transcription factor for *NHLRC2*, are essential for T-cell homeostasis (5). These findings may provide supporting evidence for the use of glucocorticoids in treating FINCA syndrome. The elevated p-ANCA found in one of our patients also indicates the involvement of an autoimmune mechanism in FINCA syndrome.

## Conclusions

We report two Chinese siblings with FINCA syndrome carrying a novel variant of *NHLRC2* gene. Systemic glucocorticoids proved effective in treating ILD in one patient, indicating that anti-inflammatory therapy may be beneficial for treating FINCA-induced ILD.

## Data Availability

The datasets for this article are not publicly available due to concerns regarding participant/patient anonymity. Requests to access the datasets should be directed to the corresponding author.
